# July 2021 civil unrest: South African diagnostic radiography students’ experiences

**DOI:** 10.4102/hsag.v28i0.2253

**Published:** 2023-08-31

**Authors:** Kathleen Naidoo, Shantel Lewis, Hafsa Essop, Gerhardus G.V. Koch, Thandokuhle E. Khoza, Nape M. Phahlamohlaka, Nicole R. Badriparsad

**Affiliations:** 1 Department of Medical Imaging and Therapeutic Sciences, Faculty of Health and Wellness Sciences, Cape Peninsula University of Technology, Cape Town, South Africa; 2 Department of Medical Imaging and Radiation Sciences, Faculty of Health Sciences, University of Johannesburg, Johannesburg, South Africa; 3 Department of Radiography, Faculty of Healthcare Sciences, University of Pretoria, Pretoria, South Africa; 4 Department of Radiography, Faculty of Health Sciences, Durban University of Technology, Berea, South Africa; 5 Department of Clinical Sciences, Faculty of Health and Environmental Sciences, Central University of Technology, Bloemfontein, South Africa; 6 Department of Radiography, Faculty of Health Sciences, Nelson Mandela University, Gqeberha, South Africa

**Keywords:** civil unrest, lived experiences, undergraduate diagnostic radiography students, clinical training, health care education work-integrated learning

## Abstract

**Background:**

South Africa (SA), in 2021, experienced a wave of civil unrest following political events that led to mass looting and the destruction of property. Civil unrests, among other disruptions, have been seen to cause ripple effects on healthcare education, particularly for radiography students who undergo work integrated learning within hospitals and universities, even during these times of unrest.

**Aim:**

This study aimed to explore and describe the undergraduate diagnostic radiography students’ experience of the civil unrest that occurred in SA in 2021.

**Setting:**

The study was conducted across five universities in South Africa, offering the diagnostic radiography programme.

**Methods:**

A qualitative, interpretive phenomenological design was employed as it enabled the researchers to facilitate focus group interviews to gain insight into the lived experiences of the students during this time.

**Results:**

Four themes emerged from the study data, namely: (1) Negative effects on students’ emotional and psychological well-being, (2) Academic and clinical support mechanisms during disruptions, (3) The influence of disruptions on clinical training, (4) Recommendations to support students for future disruptions.

**Conclusion:**

The participants from this study described the negative effects that the civil unrest had on their emotional and mental well-being. There is a need for increased support mechanisms during times of disruptions from universities across South Africa.

**Contribution:**

The findings highlight the ripple effects that disruptions, such as civil unrests, have on radiography students. This can assist universities to relook at their institutional support structures, in order to enhance the current support given to students across universities in times of disruptions.

## Introduction

Disruptions are known to affect continuous activities in a negative manner (Vidergar 2013:8). South Africa (SA) is no novice when being faced with disruptions, and in the past, experienced many forms thereof (Arndt et al. 2020; Chetty 2021). Examples over the recent years include a student-driven, ‘fees must fall’ campaign in 2015 and multiple union strikes for increased employee remuneration packages (Africa, Sokupa & Gumbi 2021). In July 2021, a civil unrest emerged in the KwaZulu-Natal (KZN) and Gauteng provinces, which soon spread to the rest of country (Elumalai et al. 2021; Rikhotso 2021). The civil unrest has been reported to have the most devastating effects on the innocent members of society, service-delivery and the economy as a whole (Africa et al. 2021). In contradiction, the 9 days of civil unrest resulted in approximately 354 deaths and an economic loss of over R50 billion (Africa et al. 2021; Vhumbunu 2021). Keeping in mind the fact that the civil unrest took place amidst the same time as the novel coronavirus disease 2019 (COVID-19), this period proved to be a double-edged sword for many (Allen 2021).

A presidential expert panel for the July 2021 civil unrest attributed the violent protests to multidimensional complexities: ‘hallowing-out’ of state enterprises, sustained unemployment, inequality, poverty, housing and land insecurity, corruption, state capture and discontent with COVID-19 restrictions catalysed by the incarceration of former president Jacob Zuma (Africa et al. 2021). A civil unrest can be explained as a violent public disturbance involving groups of people intent on causing immediate injury to people and damage to property (Alexander et al. 2018). The Constitution of the Republic of South Africa (1996) acknowledges the right to peaceful protest regulated by the *Regulations of the Gatherings Act 205 of 1993* (Republic of South Africa 1994:13). However, all protests should be within the rule of law and cognizant of human rights.

Although similar disruptions may propel change, it also results in social breakdown and has far-reaching consequences (Nyar & Wray 2012:43). In context of this paper, it affects the tertiary education of students still in training (Hall 2016; Sambo, Chatora & Goosen 2003; Yamada & Matsushima 2020). Studies show how difficult it is for students to deal with the indirect consequences of traumatic stressors related to their mental and emotional health during distressing times (Gollub et al. 2019; Greeff et al. 2021:79). Radiography education is one of the areas that may have been affected by civil unrest.

In SA, tertiary training in radiography is offered by eight institutions, which involves a work-integrated learning (WIL) component, consisting of work-based learning (WBL) at Health Professions Council of South Africa (HPCSA) accredited, clinical training centers (SAQA 2021). The HPCSA requires that competencies and capabilities aligned to the diagnostic radiographers’ scope of practice be achieved during WBL congruent with the South African Qualifications Authority (SAQA) criteria (HPCSA 2020; SAQA 2021). Work-integrated learning aims to strategically inspire students to embrace prospective future careers, thereby contributing to economic innovation and growth (Govender & Wait 2017:49). Students, workplace supervisors and employers, higher education institutions, as well as industry, government and community partners, can all benefit from WIL (Sattler & Peters 2012:8). Linking learning to the work role can be described as WBL. Three interrelated components have been identified, each of which made a significant contribution to learning: (1) structuring learning in the workplace; (2) offering suitable on-the-job training or learning opportunities and (3) identifying and offering pertinent off-the-job learning opportunities (Brennan & Little 1996:3).

Some of the indirect effects of the civil unrest could be aligned with the mental and emotional well-being of students working and studying during this devastating time (Gollub et al. 2019; Greeff et al. 2021). Students might have had families directly affected by the violence and looting, which caused many South Africans to be without access to medicine, medical assistance and food supplies (Mongale 2022:2; Statistics South Africa 2021). Understanding the radiography students’ experiences of a civil unrest is paramount as students’ academic success plays a pivotal role in breaking the cycles of poverty in previously disadvantaged communities (Gershenson & Hayes 2016). Therefore, education and academic success are crucial protective factors against adversity for radiography students within SA (World Bank 2018).

The present study aims to explore and describe the undergraduate diagnostic radiography students’ experiences of the civil unrest that occurred in SA in 2021. For the purpose of this study, civil unrest will be defined as public disturbance by a group or groups of people involving acts of violence that disrupts a community and requires intervention to maintain public safety (Federal Emergency Management Agency, undated; International Association of Chiefs of Police 2019:6).

## Methods

### Research design

An interpretive phenomenological study design was followed to explore and interpret students’ experiences during the civil unrest in South Africa in July 2021. Creswell (2012) stated, ‘a phenomenological study describes the common meaning for several individuals of their lived experiences of a concept or phenomenon’. In addition, Smith and Osborn (2015) affirmed that interpretive phenomenological study is about the detailed study of human life experience and is therefore well suited for this study.

### Population and sample

The target population comprised 1256 (*N* = 1551) undergraduate diagnostic radiography students from five universities offering bachelor’s degrees in diagnostic radiography in South Africa (see Table 1). The purposive and convenience sampling method was used to select the students to participate in the interviews. The logic and power of targeted sampling lie in selecting information-rich participants for the in-depth study (Patton 1990). The rationale behind using convenience sampling was to select participants within the target population who were available at a given time and were willing to participate in the interviews (Etikan, Musa & Alkassim 2016:2). A sample of 23 radiography students from five universities. (*n* = 23) participated in the focused group interviews. The breakdown of the sample size interviewed from each university is described in Table 1.

**TABLE 1:** Study population and sample of interviewed research participants from universities in South Africa.

Universities	Population (*N*)	Sample interviewed (*n*)
University A	243	4
University B	295	4
University C	263	4
University D	500	7
University E	250	4

**Total**	**-**	**23**

### Data-collection process

A research method employed to collect data was focused group interviews. The data-collection period was 4 months, from August 2021 to November 2021. (PN1) A researcher coordinator was appointed at each participating university, who was responsible for recruiting students during face-to-face or online classes with students. Students were asked to provide their name and contact details if they were interested in partaking in the study. Information letters were then distributed to these students through email, which detailed the aims and objectives of the study. The letter also contained the contact details of the research coordinator. A date and time that was suitable for the students were identified. One researcher facilitated the focus group interviews for all participating universities to ensure consistency in the interview process. The online interview platform was Zoom meetings and the students were emailed the link prior to the interview. The focus group interviews were audio-recorded using the recording feature on the online platform.

### Data analysis

**FIGURE 1:**
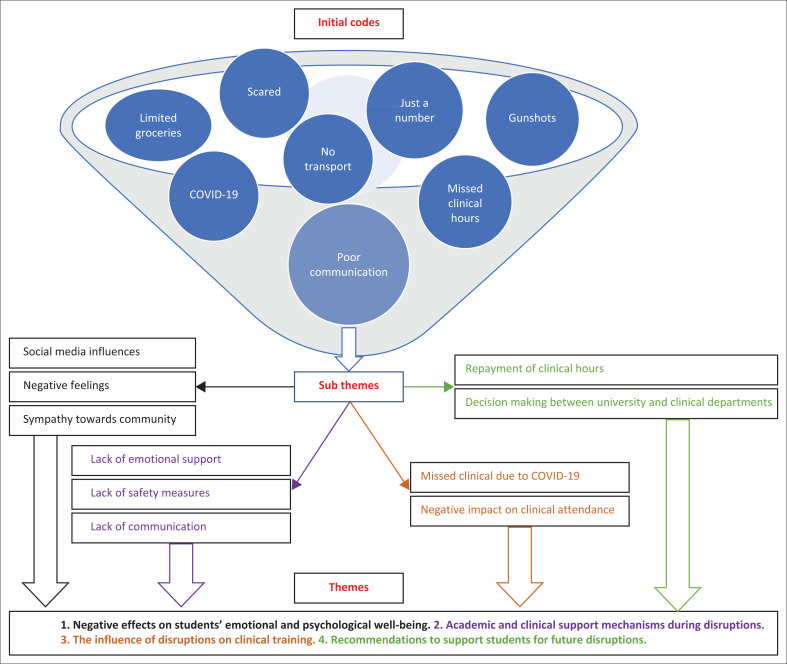
Overview of the data-analysis process of this study compiled by the authors.

Thematic analysis of data was used to identify themes in the findings. Two researchers were responsible for analysing the data to ensure consistency of the analysis process. The preliminary findings were then provided to all the researchers to check for accuracy of the interpretation. This formed part of triangulation of the data analysis process. According to Creswell and Plano Clark (2011:129), there are five steps to be followed in thematic data analysis, namely: (1) preparing the data for analysis, (2) exploring the data, (3) analysing the data, (4) representing the data for analysis and (5) validating the data. Qualitative data analysis commenced by organising documents, audio data and transcribing the data. The next process was to explore the data, which was performed by reading through the data, writing memos and developing a qualitative codebook. The actual analysis of the data would occur by coding the data, assigning labels to codes, grouping the codes into sub-themes and interrelating sub-themes into themes. The final step was presenting the findings in discussions of themes and presenting visual models, figures and tables, as demonstrated in Figure 1. In summary, Figure 1 provides a graphical representation of the data-analysis process that was followed to develop descriptive themes. In Figure 1, the initial codes represent a sample of the numerous codes, which were extracted directly from the transcripts. These codes were combined and reduced to form sub-themes, which was further reduced into themes.

### Trustworthiness

Lincoln and Guba’s (1985) criteria of credibility, dependability, transferability and confirmability were used to ensure the trustworthiness of the data collected during this research study (Lincoln & Guba 1985). Credibility was ensured whereby the themes identified were shared with the participants to determine if they recognised their experiences. Dependability and transferability were established through a detailed description of the research methodology and by using direct quotations. An audit trail detailing the data-collection process, the data analysis, interpretation and reflexivity, ensured confirmability. Triangulation of the data interpretation was achieved by all researchers viewing it and confirming its accuracy.

### Ethical clearance

Ethical clearance for the study was obtained from all the participating institutions across SA. All the participants agreed to participate by signing an informed consent form. In addition, the participants signed a second informed consent form for their interviews to be audio recorded. No identifiable details were requested or used from the participants, thus ensuring anonymity and confidentiality. The legal and ethical requirements of the *Protection of Personal Information Act* (POPIA) were adhered to throughout the research process, whereby participants voluntarily provided their details to the researchers.

Each institution recruited their own participants. The focus group interviews were scheduled at a time that was most convenient for the participants. Participation was voluntary and the participants were allowed to withdraw from the study at any time and without any penalty or consequence. Throughout the data-collection process, two researchers were responsible for conducting the interviews. During the interviews, students were encouraged to speak freely and without fear. In combination, this eliminated the power imbalance of the lecturer–student relationship. Provisions were made for those students who may have experienced emotional stress during and after their interviews, by referring them for counseling at their respective institutions.

The study involved five institutions. Ethical approval was granted by each institution University of Pretoria (UP), Faculty of Healthcare Science, Research Ethics Committee (495/2021); Cape Peninsula University of Technology (CPUT), Faculty of Health and Wellness Sciences, Research Ethics Committee (CPUT/HW-REC 2021/H38); University of Johannesburg, Faculty of Health Sciences Research Ethics Committee (REC-1230-2021); Durban University of Technology Institutional Ethics Committee (IREC 205/21); Central University of Technology (17/2021).

## Findings and discussion

The exploration of radiography students’ experiences during the July 2021 civil unrest resulted in four major themes developing from the data (Figure 1), namely: (1) Negative effects on students’ emotional and psychological well-being. (2) Academic and clinical support mechanisms during disruptions. (3) The influence of disruptions on clinical training. (4) Recommendations to support students for future disruptions. While the civil unrest mainly affected the larger cities in South Africa, there seemed to be a ‘ripple effect’ to other provinces. This in turn affected radiography students directly, if they were in the main cities, or indirectly when their family and friends were affected by various disruptions. The data presented indicate students’ feelings of distress, fear and concern over their studies, communities and family members. There was also an unmistakable need for increased support mechanisms for radiography students who experience such traumatic events. Participants provided several recommendations that they felt could assist them in the future should another disruption occur. Detailed descriptions of the participants’ feelings and experiences are provided further in the text.

### Theme 1: Negative effects on students’ emotional and psychological well-being

A traumatic and disturbing environment was described, with students expressing feelings of being scared as they could hear gunshots around them. Participants related encounters of traveling to and from their clinical placements and witnessing vehicles being burnt and stoned in front of them. Participants explained that social media had a negative effect on their mental health. The stories posted on social media of buildings and cars being destroyed during the unrest further instilled fear in the participants. Many students expressed feelings of being overwhelmed and anxious after watching the news and reading posts on Instagram. These experiences greatly impacted their ability to concentrate at work or study while at home. This is shown in the following quotations:

‘We were not in the right state of mind because a lot was going on, such as a shortage of food, and hearing gunshots.’ (University 3, Participant 4)

‘I felt so miserable, I was worried that I am in my final year, and what if I’m not able to go to com-serve next year. I couldn’t study properly because I was very worried. I ended up losing motivation to study because I thought “why waste my time studying if I’m not even going to complete this academic year.”’ (University 3, Participant 1)

‘It was quite scary I am not going to lie, so we had exams during that time, and I remember we were studying for Anatomy, we were writing Anatomy on the 15th of July. I tried not to, but I kept on going into Instagram and hearing all of this News and I just could not focus, it was so crazy what was happening, and you did not know when it was going to come to you.’ (University 4, Participant 2)

Studies in America correlate poor student performance with episodes of civil unrest or violence perpetuated by disrupted academics (Beland & Kim 2016; Gershenson & Hayes 2016; Sharkey et al. 2014). Peace and tranquility are significant to students’ academic performance and success (Omodan 2020). This is in keeping with the participants expressing the inability to concentrate because of the disruptions taking place. Social media also impacted the well-being of the students. Barret and Chen (2021:n.p.) assert:

Other factors identified as predictive of unrest are digital access and social media penetration, suggesting that the ability to communicate and coordinate on a large scale might be essential to protest activity.

Activists and protestors have used social media to attract global support for fights against a range of injustices however, during the civil unrest, social media was touted as a ‘double edge sword’ being used to weaponise the unrest (Allen 2021). During COVID-19, social media also presented the duality of information and misinformation associated with the challenge of distinguishing fake news (Venegas-Vera, Colbert & Lerma 2020).

A strong emotion also shared by many participants was the feeling of sympathy towards their communities. They witnessed businesses owned by family and friends being burnt down or getting robbed. They expressed a sense of sadness for these individuals and they worried how they would recover from this trauma. Those who had family in the midst of the unrest were worried about their livelihood as food shortages became a very real challenge. There was no stock of basic supplies such as bread and milk. Stories were also shared of the exorbitant prices of food by those who still had stock as the demand was very high. The following stories clearly demonstrate the experiences shared:

‘Personally it just made me overwhelmed and anxious. So I am originally from [*redacted for confidentiality*]. So my family in [*redacted for confidentiality*]. were really affected. They were looted like on the street and stuff. So they were like scared to leave home and like basic necessities were really expensive and I also grew up in [*redacted for confidentiality*]. So there’s a place called [*redacted for confidentiality*] in [*redacted for confidentiality*], which I saw videos of, which were really badly damaged and it made me very emotional, because that is the place where I grew up.’ (University 5, Participant 3)

I was supposed to go home that week, but I could not because the buses were not operating, and I did not have enough money. I had to wait for the situation to settle down a bit. I was very sad, and it impacted my productivity at work, I could not focus because she [*Grandmother*] was calling me constantly. She was relieved to hear that I was okay at [*redacted for confidentiality*] but I could hear the panic in her voice.’ (University 1, Participant 3)

Unrest impedes growth (Barrett & Chen 2021), evidenced by South Africa’s 1.5% GDP contraction in the third quarter of 2021 following the civil unrest (STATS SA 2021). Besides food availability, food security involves accessibility and affordability, both of which were compromised during the civil unrest (Food and Agriculture Organisation [FAO] et al. 2020:3). With over 3000 stores looted and over 40 000 businesses and informal traders affected (Warby 2021) during the protest, supply shortages were experienced country wide (Mongale 2022). South Africa is one of the world’s most unequal countries (Statistics South Africa 2020), with an ever-increasing gap between the rich and poor (Democracy Development Programme 2021). This disparity was further exacerbated by the civil unrest. A survey of healthcare professionals in South Africa revealed that:

500 healthcare professionals, two-thirds (67%) of respondents said their practice had reduced in some way during the unrest, with 6% saying they stopped practice altogether due to themselves or their staff being too scared or unable to get to the practice. Half (50%) said they had lost income due to the unrest. While only 17% considered leaving the medical profession following the unrest, 61% said it had caused them to consider leaving South Africa to work in another country. (Medical Protection Society 2021:3)

These statistics are very worrying but clearly highlight the concerns shared by the participants of this study.

### Theme 2: Academic and clinical support mechanisms during disruptions

‘South Africa can reasonably be described as the ‘protest capital of the world’ (Alexander 2016). Based on this South African situation, there is a pressing need for increased student-centred educational support mechanisms to lessen the planned and unplanned disruptions to students’ teaching, learning and training. This support should be unique to the South African context to prevent students from being subjected to violent strikes. It would be unreasonable to expect the disruptions to ease with high levels of inequality between rich and poor and high youth unemployment. The July 2021 civil unrest was one such disruption that affected students mentally, physically and emotionally. The participants of this study expressed a need for more compassion and understanding from academic staff during disruptions. There is evidence of a lack of support mechanisms in place when disruptions occur. The following quotes highlight this need:

‘The academics department should show some compassion toward students, understand the circumstances that they are faced with and try to organize transport for students who did not have money to go to clinicals.’ (University 1, Participant 1)

‘The way the university communicated with us did not help, they were not reassuring us.’ (University 2, Participant 4)

‘They should be considerate that we are students, and we are here on our own away from our families. They are not as supportive as they should be.’ (University 3, Participant 5)

‘If something of this kind happens again, our clinical coordinators should be given the power to make decisions for us when facing unforeseen circumstances.’ (University 3, Participant 5)

‘We just felt neglected as student, even the third years all of us, we were shocked at our university and how they could not stand behind us in this time.’ (University 4, Participant 2)

‘Maybe just have an online session where a lecturer would just ask the students how they are coping and just hearing if everything is okay.’ (University 4, Participant 1)

‘I know throughout the years people have said the university treats you like a number, you are just a number to them.’ (University 4, Participant 3)

These traumatic experiences of students, call for universities to find innovative interventions to support students in education, training and health during and after protest actions. According to Zhai and Du (2020), universities are responsible for supporting students and meeting their education, health and safety needs. Therefore, it means that the higher learning institutions must always be prepared to respond to disruptions not only limited to student community or union protests but also to pandemics. In such events, Reimers et al. (2020) tell us that those in charge of education must quickly devise mechanisms to support education, taking into account a particular context. For example, during the COVID-19 pandemic, university educators were compelled to rapidly adapt traditional teaching to online learning to ensure that teaching and learning continued at the university. A student-centred support structure focused on health and well-being, individual socio-economic issues and continued teaching and learning emerged as a priority.

A higher probability of depression amid and after widespread civil unrest in Hong Kong between July 2019 and July 2020 was reported (Tao et al. 2022). Similarly, another study in Hong Kong reported a high prevalence of anxiety and stress brought on by the June 2019 protests and COVID-19 (Hou et al. 2021). A study through Peru’s protracted political unrest revealed a high prevalence of mental illness, post-traumatic stress, anxiety and stressors (Tremblay, Pedersen & Errazuriz 2009). A review of 52 studies on collective action and mental health from 20 countries reported that:

[*T*]he prevalence of post-traumatic stress disorder ranged from 4% to 41% in riot-affected areas. Following a major protest, the prevalence of probable major depression increased by 7%, regardless of personal involvement in the protests, suggestive of community spillover effects. Risk factors for poorer mental health included female sex, lower socioeconomic status, exposure to violence, interpersonal conflicts, frequent social media use and lower resilience and social support. Nevertheless, two studies suggested that collective actions may reduce depression and suicide, possibly due to a collective cathartic experience and greater social cohesion within subpopulations. (Ni et al. 2020)

### Theme 3: The influence of disruptions on clinical training

Clinical training is a fundamental component in radiography education and ensures that the theory taught at the academic institutions is further enhanced and supported by real-life experiences. Disruption in training can momentously influence the overall academic performance and competency levels of the student. In this theme the effects of the civil unrest on clinical training is emphasised. While the students indicated their intention to undertake their clinical obligations, the reality of the situation posed a dire threat and they were limited by fears of their well-being and families’ concerns over their safety. Participants expressed feelings of frustration over missing their clinical training especially because they had no control over the situation. These feelings are shown in the following quotes:

‘Yes, we went to WIL for two extra weeks to cover for the lost time. It was frustrating but we had to accept it because we had no other choice.’ (University 3, Participant 2)

‘Like I feel obligated towards the university but also then my heart is going home towards my parents so yeah, I wanted to go home to be there with them knowing that I am safe, but I also did not get a choice if I wanted to go to work or not, so very obligated towards the university.’ (University 4, Participant 4)

‘My family said I should just stay at home because of the fear of the taxi war. They knew I had clinical obligations, but a lady and a driver were killed. They asked what’s the point of me going to clinical’s if I’m going to die in the process.’ (University 2, Participant 2)

‘Clinicals are a requirement therefore we as students must chin up and get it done because if we don’t, we will have to pay back the hours that we missed.’ (University 1, Participant 4)

Radiography education in South Africa was hit twofold in 2021 by the COVID-19 pandemic and the civil unrest. Clinical radiography training was adversely affected by many factors including COVID-19 hard lock down restrictions, illness and absenteeism because of contracting the virus and the civil unrest. A core consequence of the civil unrest was damage to infrastructure, a lack of safety while travelling and supply chain interferences (Makoni 2021). The healthcare sector was affected by staff shortages because of road blocks and safety concerns of being on the road. This is similar to the experiences shared by the participants of this study as they expressed fear over their safety to travel to the hospitals. According to Atiya Mosam, public health medicine specialist in Johannesburg, in order for healthcare organisations to return to its normal function, healthcare workers who were debilitated or lost their lives, would have to be replaced (Makoni 2021). This report justifies the fears expressed by the participants and their family members.

A severe implication of the unrest was the fact that students were completely helpless and were unable to meet their clinical training commitments; this forced Higher Education Institutions to remodel their academic year plan. The concurrent occurrence of the COVID-19 pandemic and the civil unrest presented a unique environment that was experienced by both academics and students for the very first time. This dual incidence gravely disrupted the clinical training of diagnostic radiography students. As a result of challenges, adjustments in the year plan was necessary to accommodate for the students’ missed clinical training. The subsequent quotes depict this:

‘When the rioting started, we couldn’t go to WIL for two weeks and that ended up disrupting our year planner because everything had to be shuffled. It was difficult to get adequate hours at WIL while also catching up on lecturers because there was Covid19 as well.’ (University 3, Participant 1)

We had three weeks of class and three weeks of WIL and during the unrest it was supposed to be our WIL, I missed out on a lot, and I am still struggling with some of the projections.’ (University 1, Participant 1)

Protest action affecting higher education institutions is not a new phenomenon; during 2015–2016, South African higher education institutions (HEI’s) were seriously affected by the Fees Must Fall protest. These protests affected the academic performance of many students and resulted in the students being unable to complete the academic year (Greeff et al. 2021). The participants of this study also shared a comparable impact of the civil unrest on the academic year planner. Amending the academic year plan was similarly reported within the field of nursing education (Agu et al. 2021) and medicine (Sharma & Bhaskar 2022). An article published by Tay, Chow and Ooi (2020) highlights concerns from stakeholders on the delayed clinical training of students and the need for preparing a contingency plan to sustain learning during similar disruptions in the future. The participants in this study revealed that even after their return to WIL, they found some of their clinical tasks to be difficult. In addition, they also shared concerns pertaining to psychological distress. Not only were the participants of this study too scared to travel to their clinical sites but also their families stepped in and cautioned them to put their own safety first. Mental health is reported as a serious and negative outcome, subsequent to a disruption, especially among healthcare professionals working in the frontline (Sharma & Bhaskar 2022).

### Theme 4: Recommendations to support students during future disruptions

The professional body for radiographers, the HPCSA mandates that students need to be assessed on clinical competency before they obtain their qualifications. One of the ways the institutions of higher education achieve this clinical placement is through hospital rotations. To ensure compliance from students with their clinical placements, institutions of higher education require students to pay back anytime that they miss through absenteeism. Based on the student’s responses, the method used to pay back time missed through absenteeism is not fair as it reduces the amount of time they spend with their families. This theme draws attention to the participant’s recommendations for future disruptions. This is based on their experiences from COVID-19 and the civil unrest. Some of these recommendations included contingency plans for compensating the missed clinical days as well as possible transport provisions. Effective communication during any disruption is vital. Proper communication will allow for a less stressful experience during these already difficult times. The students recommended communication to be timeous. This is evident in the below quotes:

‘Concerning paying back clinical time, the university should have allowed us to work on weekends because we love to spend time with our families during the holiday period.’ (University 2, Participant 4)

‘I would suggest that they provide transportation and have pick-up points at different places.’ (University 2, Participant 2)

‘When they are formulating the academic calendar they should take this into consideration, when stipulating the number of weeks for WIL they should also add two extra weeks in case is unforeseen occurrences, we can use the two weeks.’ (University 1, Participant 1)

‘The issue of communication should also be improved, they communicate with us on short notices. Communication is never done in time.’ (University 3, Participant 5)

The academic calendar in higher institutions have seen interruptions for a number of reasons, predominantly student protests being at the forefront. The contributing factors to the student protests are the quality of student accommodation and the availability of transport to campuses academic and financial exclusion, and the issues related to the National Student Financial Aid Scheme (NSFAS) (South African History Online 2021). In the event of students’ protests on campus, it also disrupts the clinical placement of students to different WIL venues. It is for this reason that students find it fitting that institutions of higher learning should strongly consider a minimum 2-week period to cater to disruptions. When clinical placements are interrupted, generally institutions of higher learning need to find means to compensate for the lost time and this includes the clinical placements extending to student holidays.

In July 2021, the country experienced a severe form of civil unrest, which led to the closure of all business activities and resulted in what could be termed as a crisis. A crisis is termed as a life-threatening event that requires a quick response from those who are involved (Palttala et al. 2012:3). The same authors add that this is when communication becomes vital as it lessens uncertainty and aims to resolve the situation. However, during the July civil unrest, students felt that communication was not coordinated resulting in different messages being sent to students. The major concern was about the suspension of WIL, that there was no clear directive and as a result there were students who continued with their WIL rotations, whereas others remained at home. Eldridge et al. emphasise that incorrect information can generate anxiety and give rise to a sense of panic. Hence, they propose that communication must be clear and consistent. It is for this reason that they recommend institutions that must identify a person responsible for the communication and team for centralised communication (Eldridge, Hampton & Marfell 2020:50).

According to O’Connor et al. (2021), expertise in communication skills, patient care and patient positioning is developed and contextualised in clinical skills laboratories and through the clinical placement of students, which can be problematic in the event of disruptions affecting universities. So, the 3D virtual reality (VR) simulation in radiography education and training is a pedagogy strategy to consider during disruptions. The VR simulation is an emerging interactive teaching strategy that has received growing attention in undergraduate medical education (Wu et al. 2022). With VR simulation, students can perform clinical practicals safely and remotely if they have a laptop and access to the platform. The sad reality about VR learning platforms is that they come at the price of purchasing student licenses, which can be costly for low-income and middle-income countries. Unfortunately, university students accessing digital devices and the internet do not make up the majority in a country such as South Africa (Reimers et al. 2020) with high socio-economic inequalities. However, this would address many of the concerns over traveling for the duration of disruptions and unsafe conditions.

## Conclusion and recommendations

This article contributes to the body of knowledge as no similar research has been conducted to date in the context of undergraduate diagnostic radiography students’ experiences during the recent civil unrest. Students shared their experiences of the civil unrest as affecting their overall well-being and clinical training. They also shared their experience of the universities and clinical training centers’ support during the civil unrest. The participants expressed a need for more compassion and understanding from academic staff and improved student support mechanisms during similar disruptions.

The participants’ lived experiences detailed fear and stress exacerbated by the media. They commiserated with their communities and relatives during this time and provided student support recommendations in anticipation of future disruptions. The recommendations range between allowing the students to work back their missed clinical time during weekends or after-hour shifts, assistance with providing additional transportation to and from the clinical sites, adding extra clinical time to the departmental year planners in preparation for the possibility of unforeseen events and also for institutions to practice timeous and effective communication with the students.

It is recommended that further research be conducted to correlate and include input from all four of the radiography categories. Similar research results may also be used to inform future educational practices, should another civil unrest be imminent. It is believed that the recommendations identified from this study can be transferred to the educational offerings of other healthcare programmes.
